# Prediction models for major adverse cardiovascular events after percutaneous coronary intervention: a systematic review

**DOI:** 10.3389/fcvm.2023.1287434

**Published:** 2024-01-08

**Authors:** Wenqi Deng, Dayang Wang, Yandi Wan, Sijia Lai, Yukun Ding, Xian Wang

**Affiliations:** ^1^ Dongzhimen Hospital, Beijing University of Chinese Medicine, Beijing, China; ^2^ Institute of Cardiovascular Diseases, Beijing University of Chinese Medicine, Beijing, China

**Keywords:** percutaneous coronary intervention, major adverse cardiovascular events, clinical predictive models, prognosis, systematic review

## Abstract

**Background:**

The number of models developed for predicting major adverse cardiovascular events (MACE) in patients undergoing percutaneous coronary intervention (PCI) is increasing, but the performance of these models is unknown. The purpose of this systematic review is to evaluate, describe, and compare existing models and analyze the factors that can predict outcomes.

**Methods:**

We adhered to the Preferred Reporting Items for Systematic Reviews and Meta-Analyses (PRISMA) 2020 during the execution of this review. Databases including Embase, PubMed, The Cochrane Library, Web of Science, CNKI, Wanfang Data, VIP, and SINOMED were comprehensively searched for identifying studies published from 1977 to 19 May 2023. Model development studies specifically designed for assessing the occurrence of MACE after PCI with or without external validation were included. Bias and transparency were evaluated by the Prediction Model Risk Of Bias Assessment Tool (PROBAST) and Transparent Reporting of a multivariate Individual Prognosis Or Diagnosis (TRIPOD) statement. The key findings were narratively summarized and presented in tables.

**Results:**

A total of 5,234 articles were retrieved, and after thorough screening, 23 studies that met the predefined inclusion criteria were ultimately included. The models were mainly constructed using data from individuals diagnosed with ST-segment elevation myocardial infarction (STEMI). The discrimination of the models, as measured by the area under the curve (AUC) or C-index, varied between 0.638 and 0.96. The commonly used predictor variables include LVEF, age, Killip classification, diabetes, and various others. All models were determined to have a high risk of bias, and their adherence to the TRIPOD items was reported to be over 60%.

**Conclusion:**

The existing models show some predictive ability, but all have a high risk of bias due to methodological shortcomings. This suggests that investigators should follow guidelines to develop high-quality models for better clinical service and dissemination.

**Systematic Review Registration:**

https://www.crd.york.ac.uk/PROSPERO/display_record.php?RecordID=400835, Identifier CRD42023400835.

## Introduction

As global populations continue to grow and age, cardiovascular diseases, particularly coronary artery disease (CAD), have emerged as significant contributors to both mortality and disability (1). Since its first introduction in 1977, percutaneous coronary intervention (PCI) techniques have seen rapid advancements. Presently, PCI is recommended for various scenarios including single-vessel disease accompanied by symptoms and ischemia, as well as early invasive treatment for acute coronary syndrome (ACS), specifically in high-risk patients (2). Despite successful revascularization, patients still face an incidence rate of approximately 20% (3) for cardiovascular events or deaths, primarily occurring within the first year after PCI (4). This has a profound impact on their prognosis and quality of life.

Major adverse cardiovascular events (MACE), which are typically defined as a combination of cardiovascular mortality, non-fatal myocardial infarction, and non-fatal stroke (3-point MACE), represent a frequently utilized outcome in cardiovascular research. When hospitalization for heart failure is also considered, it is referred to as the 4-point MACE (5). In certain studies, the scope of the MACE definition may extend to encompass additional events such as unplanned coronary revascularization, hospitalization for chest pain, arrhythmia, all-cause mortality, and others (6, 7).

Numerous studies are dedicated to pinpointing predictive factors associated with MACE occurrence. Some modifiable clinical parameters and laboratory markers have garnered attention. Indicators such as increased neutrophil-to-lymphocyte ratio (8), elevated Lp(a) level combined with heightened hs-CRP (9), the monocyte to high-density lipoprotein ratio, and Gensini score (10) are acknowledged as potential predictors of MACE in individuals undergoing PCI.

Models combined with multiple predictors may assist in identifying high-risk populations. Several predicting models (11–14) have been developed, manifesting as risk score systems or nomograms, to forecast 30-day MACE, 1-year MACE, or longer-term outcomes following PCI. However, the model performance of these models remains uncertain. The purpose of this study is to identify, describe, and appraise existing models used to predict MACE among post-PCI patients.

## Materials and methods

This study was conducted in adherence to the guidelines provided by the Preferred Reporting Items for Systematic reviews and Meta-analysis (PRISMA) 2020 (15). The study was registered at PROSPERO with CRD42023400835. Since that this review comprises published studies and publicly available data, ethical approval is not deemed necessary.

### Eligibility criteria

All model development studies, whether validated or not, whether the patient underwent elective or emergency PCI, are encompassed within the scope of this review. These models should have postoperative MACE as their designated outcome, and they are eligible for inclusion irrespective of the duration of follow-up. It is imperative that these studies report on the performance of the models, including but not limited to discrimination and calibration.

Studies will be excluded if they fall within the following categories: (1) Conference abstracts, editorials, expert views, notes, or letters; (2) Review or meta-analysis articles; (3) Full-text articles were not available; (4) Studies that developed CPMs exclusively for specific populations, such as patients with diabetes, chronic kidney disease, atrial fibrillation, or the elderly or women; (5) Studies that applied an existing model to a new domain or evaluated the performance of known models adding one or more new predictors; (6) Studies focused on comparing models rather than assessing their predictive capability for MACE; and (7) External validation articles lacking corresponding development data.

### Search strategy

We conducted searches across the following databases: Embase, PubMed, The Cochrane Library, Web of Science, CNKI, Wanfang Data, VIP and SINOMED on 19 May 2023. All studies published from 1977 in which year the first coronary intervention was reported up until the date of literature retrieval are entirely considered. In addition, we supplemented our initial search by manually reviewing the reference lists of identified studies, aiming to minimize the possibility of missing relevant data. The search strategy takes a combination of subject words (MeSH, ENTREE, and others) and free-text terms related to PCI, CPM, and MACE. The search terms encompass a range of expressions including “Percutaneous Coronary Intervention,” “Coronary Revascularization,” “major adverse cardiovascular event,” “cardiovascular outcome,” “MACE,” “prediction model,” “risk stratification,” “risk score,” and others. Endnote X9 software (Thomson Reuters, Philadelphia, Pennsylvania, USA) was used for document management and duplicate removal. In cases where multiple reports are derived from the same population, only the most recent study will be included. Detailed search strategies are provided in Supplementary Appendix S1.

### Selection process

The eligibility of studies was assessed independently (YW and SL) with a third investigator (XW) available to resolve any disagreements. Initially, articles seemingly unrelated to the intended research objectives were excluded based on the screening of titles and abstracts. Then, the studies meeting the criteria were included after a comprehensive full-text reading.

### Data extraction

As the Critical Appraisal and Data Extraction for Systematic Reviews of Prediction Modelling Studies (CHARMS) Checklist (16) suggests, descriptive tables were used to extract information that encompasses the following items: (1) characteristics of studies (e.g., the authorship, publication year, country, study design, participants, outcome definition); (2) characteristics of the models (e.g., the predictors, sample size, algorithms used to select the predictors, model development method, internal validation method, model evaluation metrics such as the area under the curve(AUC)/C-index for discrimination, calibration, sensitivity, specificity; details of dealing missing data; Model presentation). Data were extracted independently by two viewers (YD and WD), and the viewers cross-checked the data before analysis.

### Quality assessment

The assessment of the risk of bias and applicability was performed independently by the reviewers (WD and DW) using the PROBAST tool (17), which consists of four domains: participants, predictors, outcome, and analysis. To support the evaluation, a set of 20 questions (Supplementary Appendix S2) was answered with “yes,” “probably yes,” “no,” “probably no,” or “no information.”

### Data synthesis and analysis

The key findings were summarized in a narrative manner and presented in a tabular format or graphs. In addition, we analyzed the adherence to the TRIPOD (18) statement of each study. The 22 items (Supplementary Appendix S3) covered a range of aspects including the title, abstract, methods, results, and other information. No qualitative analysis was performed in this study.

## Results

### Study selection

**Figure 1:**
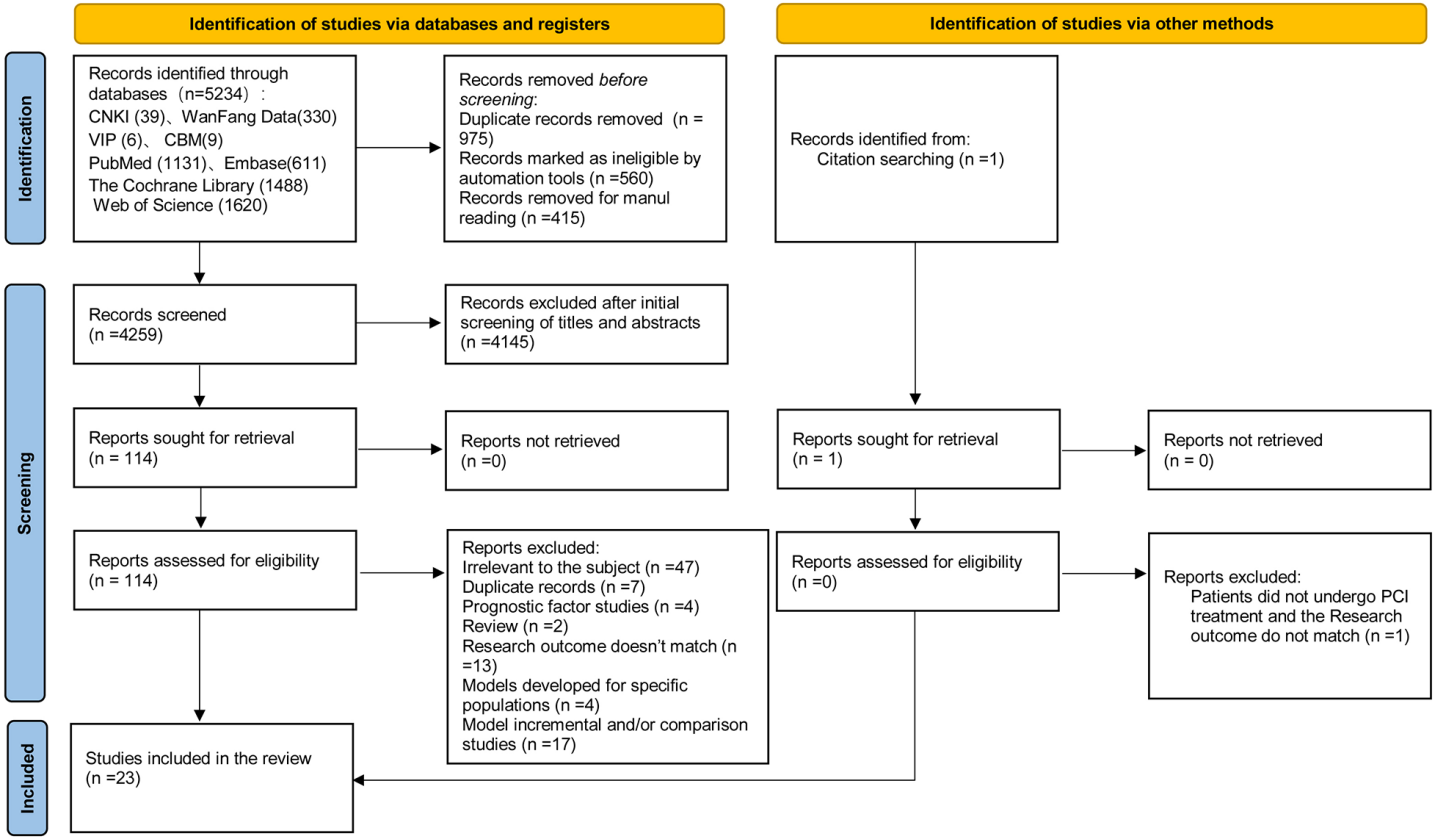
Flowchart of study inclusion.

The initial search identified 5,234 potentially relevant articles, and an additional one was added through reference citation (19). After removing 945 duplicates, 4,820 articles remained. Subsequently, we screened the titles and abstracts, resulting in the exclusion of 4,146 studies that did not meet the inclusion criteria. In total, 114 articles were reviewed in full text. Following this comprehensive evaluation, we identified 23 studies for the final analysis. Among them, 11 studies were published in Chinese (20–30), while the remaining 12 studies were published in English (11–13, 31–39). The selection process is visually depicted in Figure 1.

### Study and model characteristics

A total of 23 studies developed 28 new models for predicting MACE. Each study presented at least one model. Notably, four studies (24, 26, 32, 34) created multiple predictive models, incorporating distinct variables. The models sharing identical predictor variables and stemming from the same study population, yet predicting MACE at varying time intervals, were categorized as one model. A total of 12 studies addressed prognostic models for patients with ST-segment elevation myocardial infarction (STEMI) (20, 26–29, 32–34, 36–39). Six studies were centered on patients with myocardial infarction (MI) (11, 12, 21, 22, 24, 35). Meanwhile, three studies specifically focused on patients with acute coronary syndrome (13, 23, 25). Notably, Grayson et al. (31) recruited patients undergoing PCI across a spectrum of conditions including stable angina, unstable angina, acute myocardial infarction (AMI), and cardiogenic shock. He (30) did not explicitly mention the diagnostic information of the patients.

The prognostic models were mostly developed within 3 years (*n* = 19, 82.6%). Among the 23 studies, 20 were retrospective cohort studies, while two were prospective studies (13, 37), and one was a nested case–control study (12). Approximately 91.3% of the studies (*n* = 21) were constructed using Chinese populations, while one study (31) was based on England individuals, and another study (37) was based on Spain individuals. The models developed in the eight studies (21, 24, 25, 30, 33, 37, 39) did not undergo either internal or external validation. Three studies performed external validation by enrolling patients at different times (11, 31, 35). The study sample sizes ranged from 124 (22) to 23,718 (35). Events were reported in 21 studies (91.3%), ranging from 17 (34) to 2,615 (35). The main characteristics of the included studies are summarized in Table 1.

**Table 1:** Main characteristics of the included studies.

Study	Research type	Design	Research time	Sample	Events	EPV
For STEMI patients						
Ma J-2022	E + IV1 + IV2	R	2015.01–2017.12	554	78	13
Cui L-2022	E + IV2	R	2017.01–2019.04	354	144	18
Shi S-2022	E	R	2015.06–2019.06	500	85	8.5
Wang Y-2022	E + IV	R	2013.08–2018.07	875	292	58.4
Zhang X-2022	E + IV2	R	2017.01–2018.12	166	62	12.4
Fang C-2022	E + IV2	R	2018.01–2022.06	466	127	31.35
Marcos-Garcés-2022	E	P	2007–2017	1,118	216	54
Yao W-2022	E + IV	R	2016.01–2016.12	526	70	11.7
Yu J-2022	E	UN	2017.10–2019.12	373	UN	NI
Ma Q-2021	E + IV1	R	2017.04–2018.12	157	17	5.67
Zhao E-2020	E + IV1	R	Dryad digital Repository	460	118	11
Zhao X-2020	E	R	2010.01–2018.07	3,708	397	33.08
For AMI/MI patients						
Li Q-202	E	R	2019.01–2019.07	962	122	24.4
Zeng W-2022	E + IV	R	2018.01–2020.12	124	41	6.8
Cao J-2021	E	R	2017.06–2019.03	297	102	17
Pan D-2021	E + IV2	R	2015.01–2020.12	1,958	421	52.625
Wu C-2021	E + IV2	R	2011	23,718	2,615	290.1
	V3	P	2012.12–2014.08			
	V3	R	2015			
Zhao X-2020	E + V3	R	A:2010.01–2017.06 B:2017.07–2018.12	4,103	544	49.45
For ACS patients						
Huang G-2022	E + IV2	R	2018.09–2021.06	200	52	10.4
Li Y-2022	E	R	2019.06–2021.06	276	73	12.2
Kong S-2021	E + IV1	P	2013.01–2019.07	1,986	297	49.5
For other patients						
Grayson AD-2006	E + IV2 + V3	R	A:2001.8.1–2003.12.31 B:2004.1.1–2004.12.31	–	UN	NI
He H-2023	E	R	2018.01–2021.06	238	49	8.2

E, estimation; V, validation; IV, internal validation; IV1, randomized split validation; IV2, bootstrapping; V3, time validation; P, prospective; R, retrospective; UN, unclear; NI, no information; A, training set; B, validating set; STEMI, ST-elevation myocardial infarction; AMI, acute myocardial infarction; ACS, acute coronary syndrome; EPV, events per variable.

### Model development and performance

All models employed regression analysis, with 15 studies (12, 20–25, 27, 28, 30, 31, 34–36, 38) using logistic regression, while eight studies (11, 13, 26, 29, 32, 33, 37, 39) using Cox regression. The models were ultimately presented in various forms, such as formulas (22, 30, 31), risk scores (35, 37), nomograms (12, 13, 20, 21, 25–29, 36, 38, 39), or combinations thereof. Detailed information regarding the modeling methods, variable selecting methods, calibration method, and model presentation can be found in Table 2. The predicted outcomes spanned from in-hospital MACE (20, 21, 25, 27, 31, 35, 36) to MACE occurring post-discharge, with follow-up periods extending up to a maximum of 5 years (11). In terms of calibration, one study (29) reported Hosmer–Lemeshow test results, while six studies (11, 13, 20, 25, 28, 32, 35) provided calibration curves. In addition, nine studies (12, 21, 26, 27, 30, 31, 34, 36, 38) reported both calibration curves and test results. A total of 7 studies (22–24, 30, 33, 37, 39) did not report calibration information. The predictors and definitions of MACE across these studies were outlined in Supplementary Table S1.

**Table 2:** Model methods.

Study	Variable screening method	Modeling method	Calibration method	Presentation
For STEMI patients				
Ma J-2022	Univariate analysis	COX	Hosmer–Lemeshow test, Calibration curve	Nomogram
Cui L-2022	Lasso	Logistics	Calibration curve	Nomogram
Shi S-2022	Univariate analysis	Logistics	Hosmer–Lemeshow test, Calibration curve	Nomogram
Wang Y-2022	Univariate analysis	Logistics	Calibration curve	Nomogram
Zhang X-2022	Lasso	COX	Hosmer–Lemeshow test	Nomogram
Fang C-2022	Lasso	Logistics	Hosmer–Lemeshow test, Calibration curve	Nomogram
Marcos-Garcés-2022	Lasso	COX	NI	Risk score
Yao W-2022	Univariate analysis	Logistics	Hosmer–Lemeshow test, Calibration curve	Nomogram
Yu J-2022	Univariate analysis, step forward	COX	NI	Nomogram
Ma Q-2021	Univariate analysis, lasso	Logistics	Hosmer–Lemeshow test, Calibration curve	Nomogram, Formula
Zhao E-2020	Backward stepwise selection, AIC	COX	Calibration curve	Nomogram, Formula
Zhao X-2020	Univariate analysis, backward stepwise	COX	NI	Risk score, Nomogram
For AMI/MI patients				
Li Q-202	Lasso	Logistics	NI	Nomogram, Formula
Zeng W-2022	Univariate analysis	Logistics	NI	Formula
Cao J-2021	Lasso	Logistics	Hosmer–Lemeshow test, Calibration curve	Nomogram
Pan D-2021	Univariate analysis	Logistics	Hosmer–Lemeshow test, Calibration curve	Nomogram
Wu C-2021	Stepwise, multivariable	Logistics	Calibration curve	Risk score
Zhao X-2020	Univariate analysis, lasso	COX	Calibration curve	Risk score, Nomogram
For ACS patients				
Huang G-2022	Univariate analysis	Logistics	NI	Nomogram, Formula
Li Y-2022	Lasso	Logistics	Calibration curve	Nomogram
Kong S-2021	Univariate analysis, Forward stepwise selection, AIC	COX	Calibration curve	Nomogram
For other patients				
Grayson AD 2006	Forward stepwise	Logistics	Calibration curve, Hosmer–Lemeshow test	Formula
He H-2023	Univariate analysis	Logistics	NI	Formula

NI, no information; UN, unclear; AIC, Akaike information criterion.

**Figure 2:**
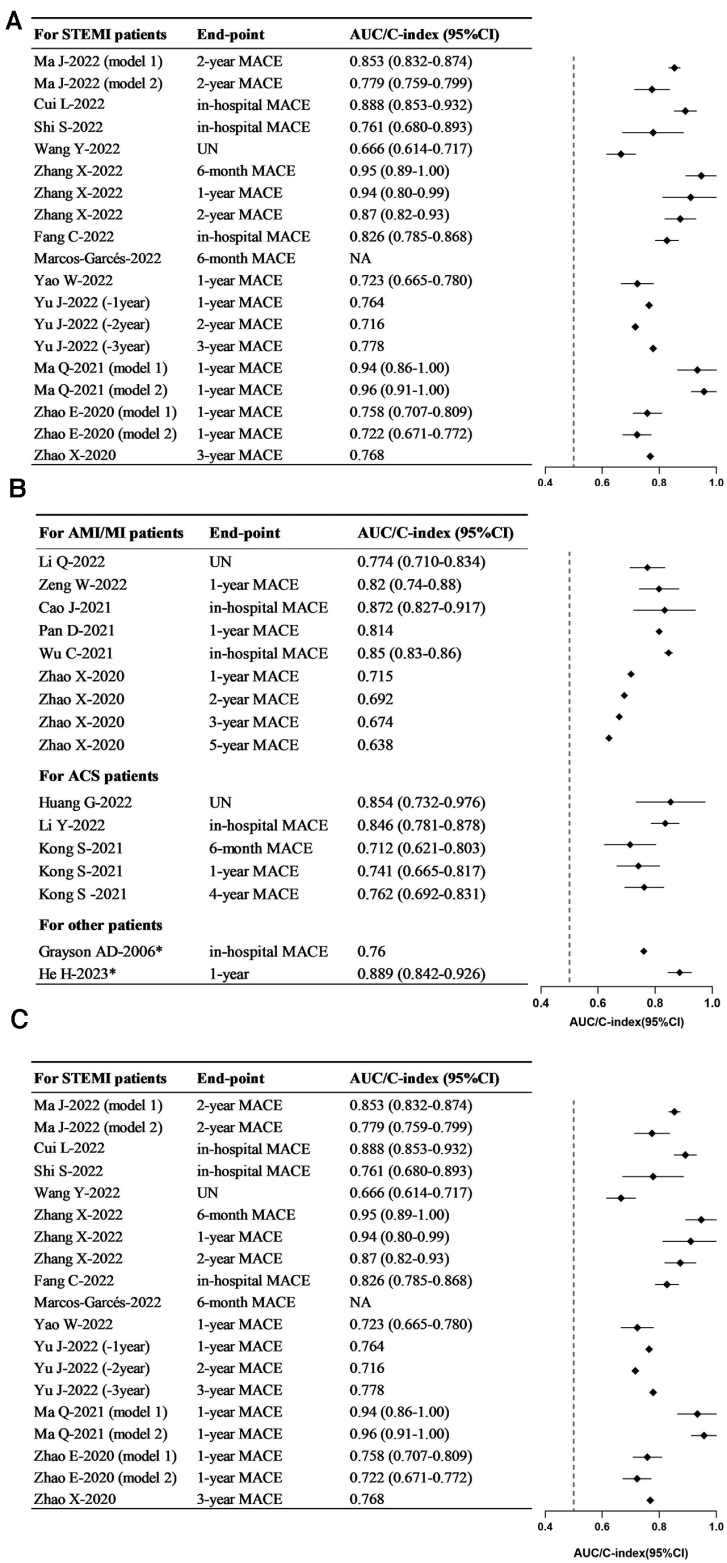
Forest plots in the discrimination of models. (**A**) Forest plot for the training set. (**B**) Forest plot for the training set—continued. (**C**) Forest plot for the validating set. STEMI, ST-segment elevation myocardial infarction; AMI, acute myocardial infarction; MI, myocardial infarction; ACS, acute coronary syndrome; MACE, major adverse cardiovascular events.

Discrimination, assessed by the area under the curve or C-index, stands as the most critical metric for evaluating model predictive performance. With the exception of one study (37), 22 studies reported model discrimination ranging from 0.638 to 0.96 (Figure 2). The AUC values for the models constructed for STEMI patients, AMI/MI patients, and ACS patients ranged from 0.666 to 0.96 (Figure 2A), 0.638 to 0.872 (Figure 2B), and 0.712 to 0.854 (Figure 2B), respectively. In the training set, the AUC values ranged from 0.72 to 0.94 (Figure 2C).

### Predictor variables

**Figure 3:**
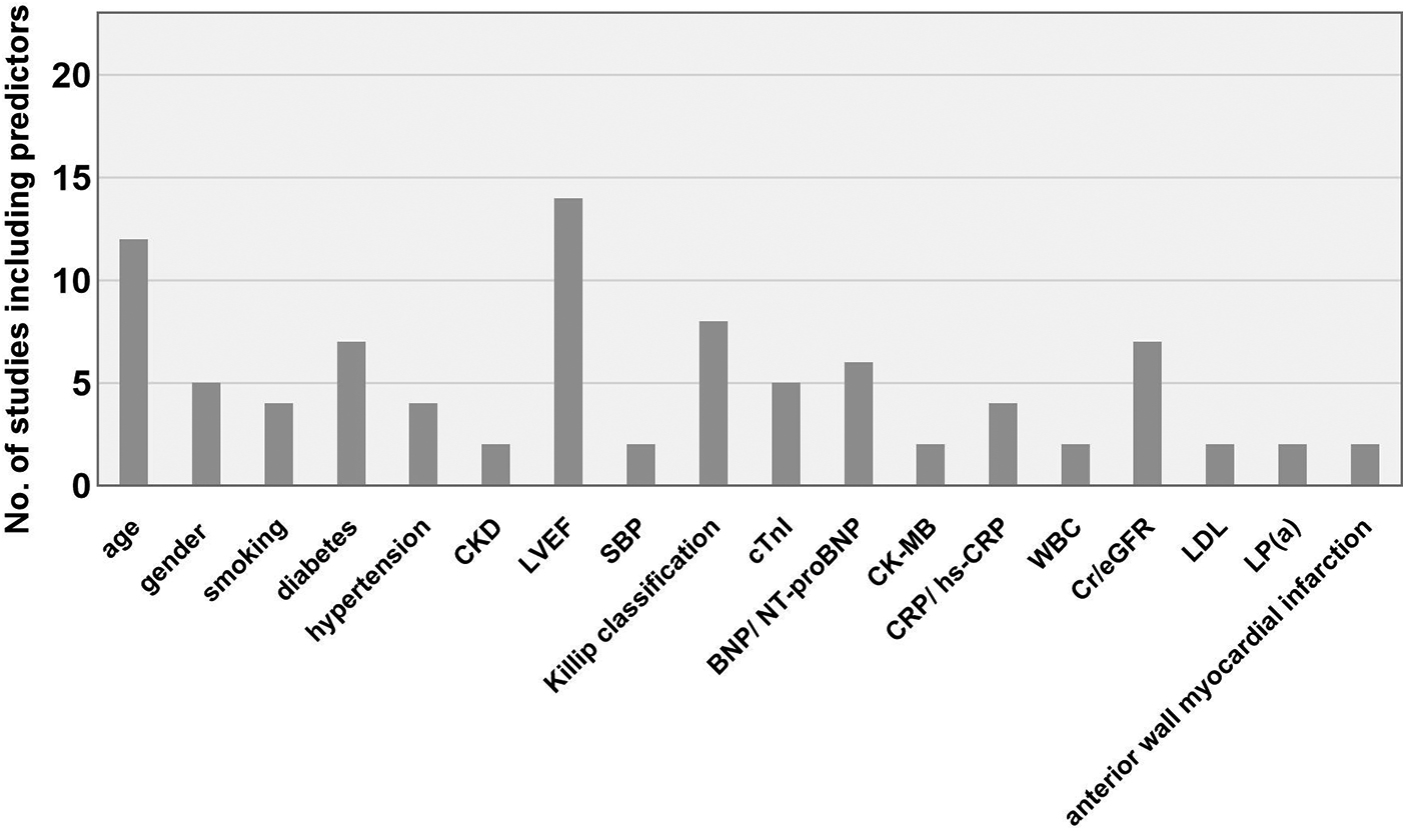
Main predictors of the included models. CKD, chronic kidney disease; LVEF, left ventricular ejection fraction; SBP, systolic blood pressure; BNP, brain natriuretic peptide; NT-proBNP, N-terminal pro-B-type natriuretic peptide; CK-MB, creatine kinase-MB; CRP, C-reactive protein; hs-CRP, high-sensitivity C-reactive protein; WBC, white blood cell count; LDL, low-density lipoprotein; eGFR, estimated glomerular filtration rate; Lp(a), lipoprotein(a).

The predictors were consistently standardized across studies before analysis. In various studies, the same indicators may be measured by different methods or at different time points. Some indicators, although different, share close clinical significance. In these cases, these indicators were analyzed collectively. For instance, terms such as “age” and “advanced age” were standardized as “age.” Similarly, variations such as “male,” “female,” “sex,” “female gender,” and “female sex” were standardized as “gender.” Expressions such as “Diabetes mellitus,” “diabetes,” “history of diabetes,” and “history of Diabetes mellitus (DM)” were all standardized as “diabetes.” Likewise, terms such as “Killip grade II–IV,” “Killip grade ≥ 3,” “Killip class,” “the Killip classification,” and “Killip's classification > I” were standardized as “Killip classification.” Similarly, expressions such as “left ventricular ejection fraction(LVEF),” “Low LVEF,” “ejection fraction at admission,” “CMR-LVEF <40%,” and “EF” were all standardized as “LVEF.” Terms such as “smoking history” and “smoking” were both standardized as “smoking history.” Terms such as “peak cTnI,” “cTnI,” “ TnI,” and “hypersensitive troponin T” were all harmonized to “cTnI”; “ high-sensitivity C-reactive protein (hs-CRP),” “C-reactive protein (CRP),” “hs-CRP > 10 mg/L,” and “hs-CRP level” were combined as “CRP/hs-CRP”; “N-terminal pro-B-type natriuretic peptide (NT-proBNP),” “baseline NT-proBNP,” “B-type natriuretic peptide (BNP),” and “BNP level” were combined as “BNP/NT-proBNP.” In addition, “creatinine” and “Scr” were both standardized as “Cr” and jointly analyzed alongside “estimated glomerular filtration rate eGFR.” The predictors applied in prediction models were multifarious covering demographic indicators, medical history, medications usage, lesion locations, PCI procedures, radiological indicators, and serological biomarkers. Most predictors appeared only once. In the same research where multiple models were constructed, the predictors were not counted repeatedly. The 10 most frequently occurring predictor variables in this review were as follows (Figure 3): LVEF (14/23), age (12/23), Killip classification (8/23), diabetes (7/23), Cr/eGFR (7/23), BNP/NT-proBNP (6/23), gender (5/23), cTnI (5/23), smoking (4/23), hypertension (4/23), and CRP/hs-CRP (4/23).

### Risk of bias and applicability assessment

**Figure 4:**
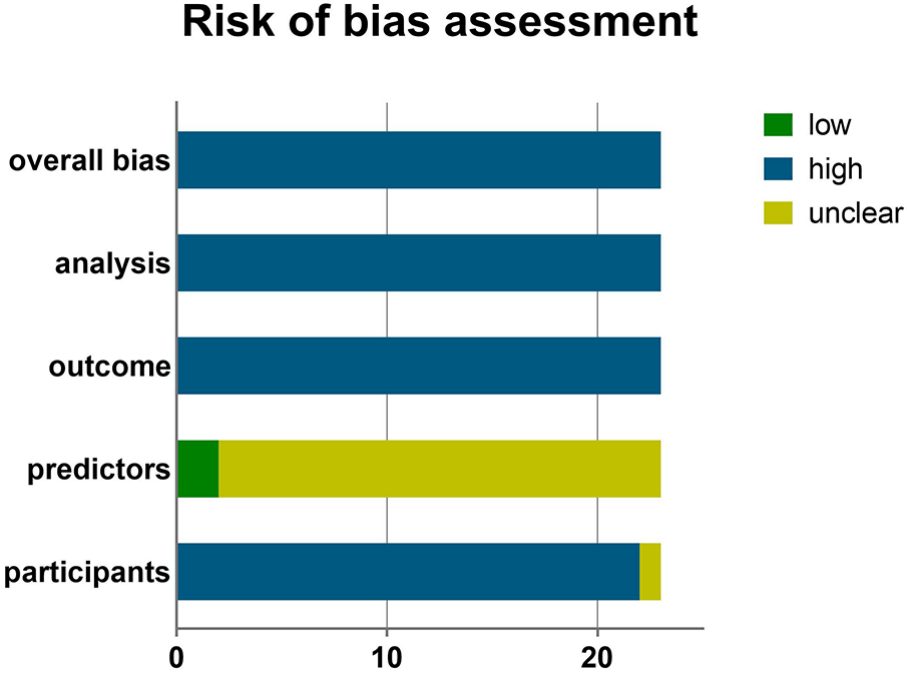
PROBAST risk of bias assessment.

According to the PROBAST assessment results, it was determined that all studies had a high risk of bias (Figure 4) owing to deficiencies in their study design, execution, and analysis. The participants' domain mostly consisted of data (*n* = 20, 86.9%) derived from retrospective cohort studies or registry data that were not originally collected for the specific purpose of developing predictive models. A total of 16 studies directly excluded cases with incomplete data or subgroups that might have an impact on predictive outcomes. This could potentially introduce higher risk of bias. In the predictors' domain, none of the studies provided any information regarding the use of a blinding method. Consequently, they all received a response of “no information” when addressing the signaling question “Were predictor assessments made without knowledge of outcome data?” In the results' domain, a predominant issue emerged: the inappropriate definition of outcomes in all studies. The outcome definition did not exclude predictor variables in five studies (20, 21, 24, 25, 30), for instance, heart failure was included in the outcome, while LVEF or NT-proBNP were used as predictors. Moreover, certain studies (20, 23, 25, 27) aimed to predict in-hospital MACE, yet they were unable to specify the timing of parameter collection. This could result in an improper time interval between the outcomes. The analysis domain is a high-risk area for bias, primarily stemming from several factors. These include sample sizes that were insufficient to fulfill the requirement of having events per variable (EPV) of ≥20 (15/23, 65.2%), the inappropriate conversion of continuous variables into categorical variables (16/23, 69.6%), the improper handling of missing data (18/23, 78.3%), and the absence of calibration reporting (6/23, 26.1%), among other factors. Predictor selection was predominantly based on univariable analysis (12, 22, 24, 26–28, 30, 37); however, it is advisable to avoid using this method. All the studies included in this review had a low risk of applicability due to the primary focus on evaluating models and identifying potential prognostic factors, with less emphasis on participant and outcome heterogeneity. The PROBAST results for each study are shown in Supplementary Table S2.

### Transparent reporting assessment

**Figure 5:**
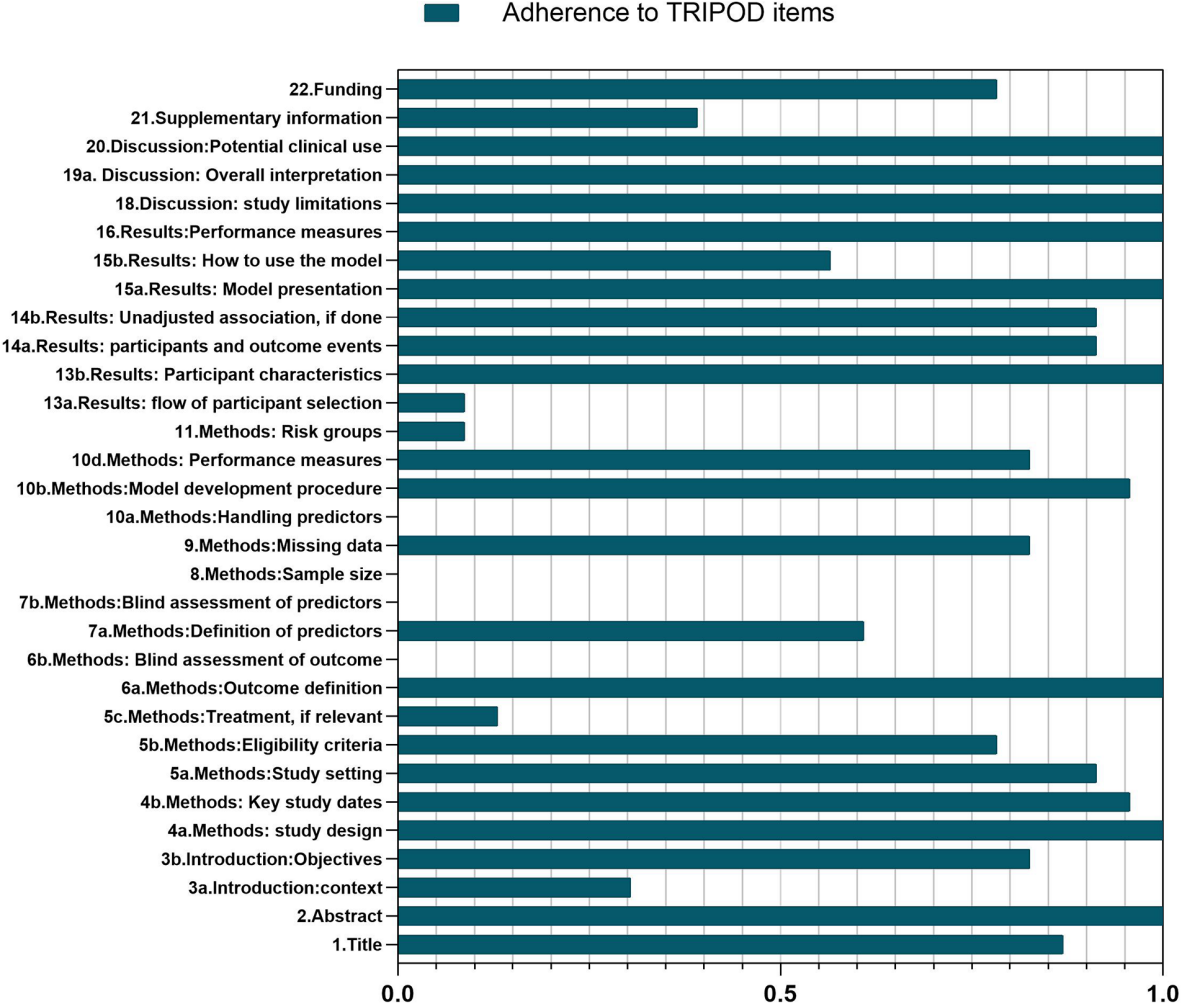
Overall adherence (%) per TRIPOD items. Total sample *n* = 23.

The study's adherence to transparent reporting of a multivariable prediction model for individual prognosis or diagnosis (TRIPOD) items reflects the completeness of reporting the included CPM studies. In this review, the median adherence rate stands at 53%, with an interquartile range (IQR) varying from 30.4% to 100% (Figure 5).

Those poorly reported items were mainly concentrated within the Methods section. The omission of blinding was a significant issue in this review since neither the evaluation of outcomes (Item 6b) nor the evaluation of predictors (Item 7b) provided any details regarding blinding procedures or measures to prevent bias. Despite the provision of sample sizes, researchers never clarified how the study size was determined (Item 8). Moreover, no studies explained how they deal with predictors (Item 10a). Five items had adherence rates below 50%. Only two studies (2/23, 8.7%) reported on risk groups (32, 35) (Item 11) and participant selection flowcharts (35, 37) (Item 13a). Furthermore, only three studies (3/23, 13.0%) reported relevant treatment (12, 25, 28) (Item 5c). In addition, seven studies (7/23, 30.4%) adhered to the recommendation citing existing models when introducing the medical background in the Introduction section (11–13, 29, 31, 32, 35) (Item 3a). Nine studies (11–13, 32–34, 36, 37, 39) (9/23, 39.1%) provided supplementary information (Item 21). Four items were incompletely reported (50%–80% adherence). The eligibility criteria (Item 5b) were addressed in 18 studies (18/23, 78.3%). Ten studies (13/23, 56.5%) described how to use the model (Item 15ba). Fourteen studies (14/23, 60.9%) detailed the definition of the predictor (Item 7a). The funding information (Item 22) was reported in 18 out of 23 articles (78.3%) where this item was suitable. All studies completely described the following nine items (100% adherence). In the Abstract, all studies provided ample information (Item 2). Within the Methods section, the description of the study design (Item 4a) and outcome definition (Item 6a) were all presented. Studies also efficiently described the characteristics of the participants (Item 13b), model presentation (Item 15a), and performance measures (Item 16) in the Results section. Meanwhile, the Discussion section of all studies were fully organized around the study limitations (Item 18), overall interpretation (Item 19a), and potential clinical application (Item 20). The articles included in this review had 80% compliance with the remaining TRIPOD items (Supplementary Figure S1).

## Discussion

Recently, there has been a noticeable increase in the number of prognostic model development studies that specifically focus on post-PCI patients. These studies frequently center on endpoints such as mortality (40–42), hemorrhage, renal injury, and atrial fibrillation (43–45). Another important endpoint that has garnered significant interest among researchers is MACE. The application of clinical prediction models can provide valuable information to patients and families and assist healthcare professionals in allocating hospital resources, potentially contributing to the improvement of healthcare quality. In addition, prediction models may aid in the clinical trial design, identifying patients with the required risk characteristics, thereby enhancing statistical power or reducing sample size and costs (46).

This systematic review provides an overview of the current landscape of models designed to predict MACE after PCI. In total, 23 articles were included in this study, presenting a total of 28 proposed models. The discriminatory power of these models varied, spanning from 0.638 to 0.96, where 66.7% of the models achieved a discrimination value exceeding 0.75. In terms of follow-up time, the AUC range for models predicting in-hospital MACE is 0.76–0.888, while the AUC range for the six models predicting 1-year MACE is 0.715–0.96. It is evident that the models explored in our study demonstrated commendable predictive powers in identifying high-risk patients.

The best-performing model (34) in this study achieved an AUC of 0.94 in the training set and an AUC of 0.90 in the validation set. This outstanding performance might be attributed to the model's reliance on a wide array of quantitative imaging parameters. Currently, polygenic risk scores, proteomics, lipidomics (47–49), and other data are gradually being introduced to predict cardiovascular events. Clearly, clinical predictive models based on easily accessible traditional risk factors may be more straightforward to apply and generalize. Collecting and utilizing such data may involve higher costs but hold the potential to improve model performance. Artificial intelligence (AI) and machine learning algorithms, including techniques such as Extreme Gradient Boosting, the Gaussian mixture model, Decision Tree, and Random Forest (50–53) have become increasingly prevalent in addressing such challenges. Machine learning with its remarkable capability to analyze extensive volumes of intricate data (54) holds tremendous potential for enhancing predictive performance and should see wider adoption in the medical field.

This review has found that the most common variables for predicting post-PCI MACE include LVEF, age, Killip classification, diabetes, Cr, BNP, gender, troponin, smoking, hypertension, and CRP. These variables encompass unmodifiable factors such as demographic data like age and gender. The included studies consistently indicate that older age is associated with worse outcomes. The odds ratio (OR) for individuals over 60 years of age is 1.212 times higher than for those under 60 years (25), while individuals aged 80 and above have a higher risk of MACE compared with those aged 70–79 (31). Several studies suggest that female patients generally have worse prognoses, although Ma et al.'s study (26) arrived at the opposite conclusion. This could be attributed to the study's male-to-female patient ratio, which was 3.5 times higher, with no adjustment made during the analysis. In the models developed for STEMI patients, the top three variables are LVEF, Killip classification, and age. For AMI patients, the most common variables include LVEF, diabetes, and age, while in ACS patients, the primary variables are LVEF, age, and BNP. There is a consistent demonstration of the value of LVEF and age for adverse outcomes of PCI patients. When analyzing these predictive factors collectively, it cannot be overlooked that the Killip classification assesses cardiac function in cases of AMI, but it is not applicable to patients with unstable angina within the ACS population. Variables assessing kidney function, such as a history of CKD, SCr, and eGFR, were also included in multiple studies. This emphasizes the need for clinical attention to both cardiac and renal function to identify individuals at risk of adverse outcomes early. In recent years, several studies have revealed the connection between inflammation and coronary heart disease. Inflammatory-related variables such as NLR, CRP, and hs-CRP were also incorporated into multiple models. Residual inflammatory risk (RIR), defined as when plasma LDL-C levels are below 1.8 mmol/L and plasma hs-CRP levels are ≥2 mg/L (55), was employed in a model constructed to predict in-hospital MACE in AMI patients after PCI (21). The model achieved an AUC value of 0.82, indicating a close correlation between RIR and recurrent cardiovascular events.

However, the reporting is not rigorous enough, and all studies were judged to be at high risk of bias. The primary factors contributing to this included retrospective study designs, the absence of blinding during the assessment of predictors or outcomes, unjustified categorization and definition of outcomes, failure to circumvent univariate analysis when screening variables, insufficient sample size for EPV, improper internal validation methods, lack of external validation, and improper handling of missing data. The PROBAST, released in 2019 (17), serves as a pivotal tool for evaluating clinical predictive modeling during systematic reviews. Intriguingly, despite 22 out of 23 articles in this review being published post-2019, they still exhibited methodological shortcomings across various facets. To enhance the quality of clinical predictive modeling and maximize the value of such models in clinical applications, it is advisable for researchers to acquaint themselves with the PROBAST tool at an early stage to minimize possible biases in the study design or data sources.

### Potential clinical applications

Although these models demonstrate good predictive performance, all studies were at high overall risk of bias. This review reveals that there is insufficient evidence to apply any of these models in clinical practice. However, clinical practitioners can pay closer attention to the common variables in the models and intervene appropriately with modifiable risk factors, which may help reduce the incidence of MACE.

### Strength and limitations

This study marks a notable effort in directing our attention towards predicting models for MACE in post-PCI patients. We adopted convincing tools such as PROBAST and TRIPOD in this systematic review to provide more informative results. Certain procedural characteristics, such as access site, vessel dilation, type of PCI, initial and final TIMI flow grade, stent type (drug-eluting stents or bare metal stents), and stent length, can reflect the contemporariness of the treatment and indicate the complexity of the medical condition (56). They can also serve as a basis for identifying high-risk individuals. However, these factors have not been sufficiently considered in the current models. Future researchers should give greater consideration to these aspects in their studies. In addition, the application of a language filter limiting studies to Chinese and English during literature screening may result in the omission of valuable data and insights published in other languages. This could introduce some and potentially impact the conclusions of this systematic review. Due to the limited number of relevant studies, we incorporated prediction models designed for patients undergoing PCI in all clinical settings. Nevertheless, it is essential to recognize that patient prognosis and risk factors exhibit variations in different clinical settings. Moreover, these studies vary in their definitions of MACE and the duration of follow-up. Although we made efforts to describe them categorically, the inherent heterogeneity makes further quantitative analysis challenging.

## Conclusion

In summary, despite the rising number of modeling studies, the practicality of many models remains uncertain due to a lack of external validation and methodological shortcomings. It is imperative for researchers to adhere to guidelines to enhance study designs and construct models with high clinical applicability using proper internal validation and external methods.

## Data Availability

The datasets used and/or analyzed during this study are available from the corresponding author on reasonable request.

## References

[1] Roth GA Mensah GA Johnson CO Addolorato G Ammirati E Baddour LM Global burden of cardiovascular diseases and risk factors, 1990–2019: update from the GBD 2019 study. J Am Coll Cardiol. (2020) 76(25):2982–3021. 10.1016/j.jacc.2020.11.010 33309175 PMC7755038

[2] Bhatt DL. Percutaneous coronary intervention in 2018. JAMA. (2018) 319(20):2127–8. 10.1001/jama.2018.5281 29800163

[3] Fanaroff AC Zakroysky P Wojdyla D Kaltenbach LA Sherwood MW Roe MT Relationship between operator volume and long-term outcomes after percutaneous coronary intervention. Circulation. (2019) 139(4):458–72. 10.1161/circulationaha.117.033325 30586696 PMC6340715

[4] Madhavan MV Kirtane AJ Redfors B Généreux P Ben-Yehuda O Palmerini T Stent-related adverse events >1 year after percutaneous coronary intervention. J Am Coll Cardiol. (2020) 75(6):590–604. 10.1016/j.jacc.2019.11.058 32057373

[5] Peng ZY Yang CT Kuo S Wu CH Lin WH Ou HT. Restricted mean survival time analysis to estimate Sglt2i-associated heterogeneous treatment effects on primary and secondary prevention of cardiorenal outcomes in patients with type 2 diabetes in Taiwan. JAMA Netw Open. (2022) 5(12):e2246928. 10.1001/jamanetworkopen.2022.46928 36520437 PMC9856417

[6] Chen MY Rochitte CE Arbab-Zadeh A Dewey M George RT Miller JM Prognostic value of combined Ct angiography and myocardial perfusion imaging versus invasive coronary angiography and nuclear stress perfusion imaging in the prediction of major adverse cardiovascular events: the Core320 multicenter study. Radiology. (2017) 284(1):55–65. 10.1148/radiol.2017161565 28290782 PMC5495129

[7] Cheng L Rong J Zhuo X Gao K Meng Z Wen X Prognostic value of malnutrition using geriatric nutritional risk index in patients with coronary chronic total occlusion after percutaneous coronary intervention. Clin Nutr. (2021) 40(6):4171–9. 10.1016/j.clnu.2021.01.042 33627243

[8] Wada H Dohi T Miyauchi K Shitara J Endo H Doi S Pre-procedural neutrophil-to-lymphocyte ratio and long-term cardiac outcomes after percutaneous coronary intervention for stable coronary artery disease. Atherosclerosis. (2017) 265:35–40. 10.1016/j.atherosclerosis.2017.08.007 28843126

[9] Yuan D Wang P Jia S Zhang C Zhu P Jiang L Lipoprotein(a), high-sensitivity C-reactive protein, and cardiovascular risk in patients undergoing percutaneous coronary intervention. Atherosclerosis. (2022) 363:109–16. 10.1016/j.atherosclerosis.2022.10.013 36357218

[10] Yu R Hou R Wang T Li T Han H An J. Correlation between monocyte to high-density lipoprotein ratio and major adverse cardiovascular events in patients with acute coronary syndrome after percutaneous coronary intervention. Pak J Med Sci. (2021) 37(3):885–9. 10.12669/pjms.37.3.3469 34104183 PMC8155411

[11] Zhao X Liu C Zhou P Sheng Z Li J Zhou J Estimation of major adverse cardiovascular events in patients with myocardial infarction undergoing primary percutaneous coronary intervention: a risk prediction score model from a derivation and validation study. Front Cardiovasc Med. (2020) 7:603621. 10.3389/fcvm.2020.603621 33330667 PMC7728669

[12] Pan D Xiao S Hu Y Pan Q Wu Q Wang X Clinical nomogram to predict major adverse cardiac events in acute myocardial infarction patients within 1 year of percutaneous coronary intervention. Cardiovasc Ther. (2021) 2021:3758320. 10.1155/2021/3758320 34987604 PMC8687843

[13] Kong S Chen C Zheng G Yao H Li J Ye H A prognostic nomogram for long-term major adverse cardiovascular events in patients with acute coronary syndrome after percutaneous coronary intervention. BMC Cardiovasc Disord. (2021) 21(1):253. 10.1186/s12872-021-02051-0 34022791 PMC8141252

[14] Mrdovic I Savic L Krljanac G Asanin M Perunicic J Lasica R Predicting 30-day major adverse cardiovascular events after primary percutaneous coronary intervention. The risk-PCI score. Int J Cardiol. (2013) 162(3):220–7. 10.1016/j.ijcard.2011.05.071 21663982

[15] Page MJ Moher D Bossuyt PM Boutron I Hoffmann TC Mulrow CD Prisma 2020 explanation and elaboration: updated guidance and exemplars for reporting systematic reviews. Br Med J. (2021) 372:n160. 10.1136/bmj.n160 33781993 PMC8005925

[16] Moons KG de Groot JA Bouwmeester W Vergouwe Y Mallett S Altman DG Critical appraisal and data extraction for systematic reviews of prediction modelling studies: the charms checklist. PLoS Med. (2014) 11(10):e1001744. 10.1371/journal.pmed.1001744 25314315 PMC4196729

[17] Wolff RF Moons KGM Riley RD Whiting PF Westwood M Collins GS PROBAST a tool to assess the risk of bias and applicability of prediction model studies. Ann Intern Med. (2019) 170(1):51–8. 10.7326/m18-1376 30596875

[18] Collins GS Reitsma JB Altman DG Moons KG. Transparent reporting of a multivariable prediction model for individual prognosis or diagnosis (tripod): the tripod statement. Br Med J. (2015) 350:g7594. 10.1136/bmj.g7594 25569120

[19] D'Ascenzo F De Filippo O Gallone G Mittone G Deriu MA Iannaccone M Machine learning-based prediction of adverse events following an acute coronary syndrome (praise): a modelling study of pooled datasets. Lancet. (2021) 397(10270):199–207. 10.1016/S0140-6736(20)32519-8 33453782

[20] Cui L Dong S Li C Yu H Han Y Song H Construction of nomogram model to predict the risk of major 356 adverse cardiovascular events in patients with St-segment elevation myocardial infarction after primary percutaneous coronary intervention. J Chin Pract Diag Ther. (2021) 35(4):367–71. 10.13507/j.issn.1674-3474.2021.04.011

[21] Cao J Zhang L Zhan L Ma L. Predictive value of residual inflammation risk-based nomogram model for major adverse cardiovascular events in acute myocardial infarction patients after percutaneous coronary intervention. J Third Mil Med Univ. (2021) 43(18):1821–30. 10.16016/j.1000-5404.202103184

[22] Zeng W Wu A Jiang J Chen R Zhang R Wang C. Influencing factors and predictive modeling of major cardiovascular events occurring 1 year after PCI in patients with acute myocardial infarction. Chin J Gerontol. (2022) 42(20):4905–8. 10.3969/j.issn.1005-9202.2022.20.001

[23] Huang G Zheng X Chen S. Major cardiovascular events in patients with acute coronary syndrome: construction of clinical prediction model based on platelet to lymphocyte ratio. S China J Cardiovasc Dis. (2022) 28(5):411–6. 10.3969/j.issn.1007-9688.2022.05.06

[24] Li Q Tan X Jiang W Yuan M Ni H Wang Y Predictive model for long-term major adverse cardiovascular events in patients with acute myocardial infarction undergoing percutaneous coronary intervention. Chin Gen Pract. (2022) 25(24):2965–74. 10.12114/j.issn.1007-9572.2022.0237

[25] Li Y Liang L Chen B Wang Y. Construction of an in-hospital mace attack risk prediction model for patients with acute coronary syndrome after percutaneous coronary intervention. Chin Heart J. (2022) 34(5):531–6. 10.12125/j.chj.202203019

[26] Ma J Liu J Bai G Liu X Li Y. Construction of a predictive model based on plasma Dpp4 activity to assess the 2-year risk of MACEs events in STEMI patients after primary PCI. Med J W China. (2022) 34(3):396–401. 10.3969/j.issn.1672-3511.2022.03.015

[27] Shi S Hou W Li Z Jia X Ren Z. Predictive value of inflammatory factors for in-hospital major 380 adverse cardiovascular events in patients with acute ST-segment elevation myocardial infarction after percutaneous coronary intervention. Chin Heart J. (2022) 34(04):422–7. 10.12125/j.chj.202110010

[28] Wang Y Yan W Xu Y Gu Y Zhang M Yang X Song Y Risk factors of major adverse cardiovascular events and establishment of nomogram model after percutaneous coronary intervention. Chin J Evid Bases Cardiovasc Med. (2022) 14(11):1386–90. 10.3969/j.issn.1674-4055.2022.11.23

[29] Zhang X Ding Y Yao Y Gu Y Zhang X. Establishment and validation of prognostic risk nomogram model among patients with premature ST-segment elevation myocardial infarction. J Nanjing Med Univ. (2022) 42(11):1539–46, 52. 10.7655/nydxbns20221106

[30] He H. Construction and analysis of risk model for major adverse cardiovascular events in 238 patients after percutaneous coronary intervention. Chin J Clin. (2023) 51(04):409–11. 10.3969/j.issn.2095-8552.2023.04.010

[31] Grayson AD Moore RK Jackson M Rathore S Sastry S Gray TP Multivariate prediction of major adverse cardiac events after 9,914 percutaneous coronary interventions in the north west of England. Heart. (2006) 92(5):658–63. 10.1136/hrt.2005.066415 16159983 PMC1860907

[32] Zhao EF Xie H Zhang YS. A nomogram based on apelin-12 for the prediction of major adverse cardiovascular events after percutaneous coronary intervention among patients with ST-segment elevation myocardial infarction. Cardiovasc Ther. (2020) 2020:9416803. 10.1155/2020/9416803 32099583 PMC7026703

[33] Zhao X Wang Y Liu C Zhou P Sheng Z Li J Prognostic value of total bilirubin in patients with ST-segment elevation acute myocardial infarction undergoing primary coronary intervention. Front Cardiovasc Med. (2020) 7:615254. 10.3389/fcvm.2020.615254 33392275 PMC7773653

[34] Ma Q Ma Y Wang X Li S Yu T Duan W A radiomic nomogram for prediction of major adverse cardiac events in ST-segment elevation myocardial infarction. Eur Radiol. (2021) 31(2):1140–50. 10.1007/s00330-020-07176-y 32856164

[35] Wu C Huo X Liu J Zhang L Bai X Hu S Development and validation of a risk prediction model for in-hospital major cardiovascular events in patients hospitalised for acute myocardial infarction. BMJ Open. (2021) 11(5):e042506. 10.1136/bmjopen-2020-042506 34045213 PMC8162080

[36] Fang C Chen Z Zhang J Jin X Yang M. Construction and evaluation of nomogram model for individualized prediction of risk of major adverse cardiovascular events during hospitalization after percutaneous coronary intervention in patients with acute ST-segment elevation myocardial infarction. Front Cardiovasc Med. (2022) 9:1050785. 10.3389/fcvm.2022.1050785 36620648 PMC9810984

[37] Marcos-Garcés V Perez N Gavara J Lopez-Lereu MP Monmeneu JV Rios-Navarro C Risk score for early risk prediction by cardiac magnetic resonance after acute myocardial infarction. Int J Cardiol. (2022) 349:150–4. 10.1016/j.ijcard.2021.11.050 34826497

[38] Yao WS Li J. Risk factors and prediction nomogram model for 1-year readmission for major adverse cardiovascular events in patients with STEMI after PCI. Clin Appl Thromb Hemost. (2022) 28:10760296221137847. 10.1177/10760296221137847 36380508 PMC9676288

[39] Yu J Liu Y Peng W Xu Z. Serum vcam-1 and icam-1 measurement assists for mace risk estimation in ST-segment elevation myocardial infarction patients. J Clin Lab Anal. (2022) 36(10):e24685. 10.1002/jcla.24685 36045604 PMC9550957

[40] Zhang J Zheng YY Wu TT Ma X Ma YT Xie X Blood routine test parameters score, a novel predictor of adverse outcomes of coronary artery disease patients with or without diabetes who underwent percutaneous coronary intervention: a retrospective cohort study. ACS Omega. (2021) 6(48):32508–16. 10.1021/acsomega.1c03990 34901600 PMC8655762

[41] Liu Q Zhang Y Zhang PQ Zhang JB Cao XJ He SS Both baseline Selvester QRS score and change in QRS score predict prognosis in patients with acute ST-segment elevation myocardial infarction after percutaneous coronary intervention. Coron Artery Dis. (2020) 31(5):403–10. 10.1097/mca.0000000000000869 32168048 PMC7331825

[42] Damman P Kuijt WJ Woudstra P Haeck JDE Koch KT Gu YLL Multiple biomarkers at admission are associated with angiographic, electrocardiographic, and imaging cardiovascular mechanistic markers of outcomes in patients undergoing primary percutaneous coronary intervention for acute ST-elevation myocardial infarction. Am Heart J. (2012) 163(5):783–9. 10.1016/j.ahj.2012.01.004 22607855

[43] Shoji S Kohsaka S Kumamaru H Nishimura S Ishii H Amano T Risk prediction models in patients undergoing percutaneous coronary intervention: a collaborative analysis from a Japanese administrative dataset and nationwide academic procedure registry. Int J Cardiol. (2023) 370:90–7. 10.1016/j.ijcard.2022.10.144 36306945

[44] Niimi N Shiraishi Y Sawano M Ikemura N Inohara T Ueda I Machine learning models for prediction of adverse events after percutaneous coronary intervention. Sci Rep. (2022) 12(1):6262. 10.1038/s41598-022-10346-1 35428765 PMC9012739

[45] Aittokallio J Kauko A Vaura F Salomaa V Kiviniemi T Schnabel RB Polygenic risk scores for predicting adverse outcomes after coronary revascularization. Am J Cardiol. (2022) 167:9–14. 10.1016/j.amjcard.2021.11.046 34998506

[46] Hizoh I Domokos D Banhegyi G Becker D Merkely B Ruzsa Z. Mortality prediction algorithms for patients undergoing primary percutaneous coronary intervention. J Thorac Dis. (2020) 12(4):1706–20. 10.21037/jtd.2019.12.83 32395313 PMC7212133

[47] Lu X Liu Z Cui Q Liu F Li J Niu X A polygenic risk score improves risk stratification of coronary artery disease: a large-scale prospective Chinese cohort study. Eur Heart J. (2022) 43(18):1702–11. 10.1093/eurheartj/ehac093 35195259 PMC9076396

[48] Helgason H Eiriksdottir T Ulfarsson MO Choudhary A Lund SH Ivarsdottir EV Evaluation of large-scale proteomics for prediction of cardiovascular events. JAMA. (2023) 330(8):725–35. 10.1001/jama.2023.13258 37606673 PMC10445198

[49] Nurmohamed NS Kraaijenhof JM Mayr M Nicholls SJ Koenig W Catapano AL Proteomics and lipidomics in atherosclerotic cardiovascular disease risk prediction. Eur Heart J. (2023) 44(18):1594–607. 10.1093/eurheartj/ehad161 36988179 PMC10163980

[50] Katsuki M Matsumori Y Kawamura S Kashiwagi K Koh A Tachikawa S Developing an artificial intelligence-based headache diagnostic model from a clinic patients’ dataset. Headache. (2023) 63(8):1097–108. 10.1111/head.14611 37596885

[51] Carvantes-Barrera A Díaz-González L Rosales-Rivera M Chávez-Almazán LA. Risk factors associated with COVID-19 lethality: a machine learning approach using Mexico database. J Med Syst. (2023) 47(1):90. 10.1007/s10916-023-01979-4 37597034

[52] Ito T Morooka H Takahashi H Fujii H Iwaki M Hayashi H Identification of clinical phenotypes associated with poor prognosis in patients with nonalcoholic fatty liver disease via unsupervised machine learning. J Gastroenterol Hepatol. (2023) 38(10):1832–39. 10.1111/jgh.16326 37596843

[53] Han S Sohn TJ Ng BP Park C. Predicting unplanned readmission due to cardiovascular disease in hospitalized patients with cancer: a machine learning approach. Sci Rep. (2023) 13(1):13491. 10.1038/s41598-023-40552-4 37596346 PMC10439193

[54] Obermeyer Z Emanuel EJ. Predicting the future—big data, machine learning, and clinical medicine. N Engl J Med. (2016) 375(13):1216–9. 10.1056/NEJMp1606181 27682033 PMC5070532

[55] Ridker PM. Clinician’s guide to reducing inflammation to reduce atherothrombotic risk: JACC review topic of the week. J Am Coll Cardiol. (2018) 72(25):3320–31. 10.1016/j.jacc.2018.06.082 30415883

[56] Szabo D Szabo A Magyar L Banhegyi G Kugler S Pinter A Admission lactate level and the grace 2.0 score are independent and additive predictors of 30-day mortality of STEMI patients treated with primary PCI-results of a real-world registry. PLoS One. (2022) 17(11):e0277785. 10.1371/journal.pone.0277785 36383629 PMC9668119

