# Supplementary material for: Prediction models for major adverse cardiovascular events after percutaneous coronary intervention: a systematic review
Source: Front Cardiovasc Med. 2024 Jan 8;10:1287434. doi: 10.3389/fcvm.2023.1287434 (PMC10800829; doi:10.3389/fcvm.2023.1287434)

Supplementary Material

# Supplementary Appendix S1. Search Strategy Conducted on May 19, 2023

# Supplementary Appendix S2. PROBAST Signaling questions

# Supplementary Appendix S3. TRIPOD Checklist

# Supplementary Appendix S4. PRISMA2020 Checklist

# Supplementary Table S1. Predictors and MACE definitions

# Supplementary Table S2. PROBAST results of each study

# Supplementary Figure S1. TRIPOD results of each study

# Supplementary Appendix 1. Search Strategy Conducted on May 19, 2023

# PUBMED

# #1 "Percutaneous Coronary Intervention"[Mesh] OR Coronary Intervention, Percutaneous OR Coronary Interventions, Percutaneous OR Intervention, Percutaneous Coronary OR Interventions, Percutaneous Coronary OR Percutaneous Coronary Interventions OR Percutaneous Coronary Revascularization OR Coronary Revascularization, Percutaneous OR Coronary Revascularizations, Percutaneous OR Percutaneous Coronary Revascularizations OR Revascularization, Percutaneous Coronary OR Revascularizations, Percutaneous Coronary

# #2 major adverse cardiovascular event OR adverse cardiovascular event OR cardiovascular event OR cardiovascular outcome OR MACE

# #3 (((prediction model[Title/Abstract]) OR (risk stratification[Title/Abstract])) OR (risk score[Title/Abstract])) OR (risk assessment[Title/Abstract])

# #4 #1 AND #2 AND #3

# Embase

# #1 ‘percutaneous coronary intervention’:ab,ti

# #2 ((((((((((coronary AND intervention, AND percutaneous OR coronary)AND interventions, AND percutaneous OR intervention,)AND percutaneous AND coronary OR interventions,)AND percutaneous AND coronary OR percutaneous)AND coronary AND intervention OR percutaneous)AND coronary AND revascularization OR coronary)AND revascularization, AND percutaneous OR coronary)AND revascularization, AND percutaneous OR percutaneous)AND coronary AND revascularization OR revascularization,) AND percutaneous AND coronary OR revascularization,)AND percutaneous AND coronary

# #3 #1 OR #2

# #4 ‘major adverse cardiovascular event’:ti,ab,kw OR ‘adverse cardiovascular event’:ti,ab,kw OR ‘cardiovascular event’:ti,ab,kw OR ‘cardiovascular outcome’:ti,ab,kw OR mace:ti,ab,kw

# #5 ‘predict* model’:ti,ab,kw OR ‘risk stratification’:ti,ab,kw OR ‘risk score’:ti,ab,kw OR ‘risk assessment’:ti,ab,kw

# #6 #3 AND #4 AND #5

# The Cochrane Library

# #1 MeSH descriptor: [Percutaneous Coronary Intervention] explode all trees

# #2 (Coronary Intervention, Percutaneous OR Coronary Interventions, Percutaneous OR Intervention, Percutaneous Coronary OR Interventions, Percutaneous Coronary OR Percutaneous Coronary Interventions OR Percutaneous Coronary Revascularization OR Coronary Revascularization, Percutaneous OR Coronary Revascularizations, Percutaneous OR Percutaneous Coronary Revascularizations OR Revascularization, Percutaneous Coronary OR Revascularizations, Percutaneous Coronary):ti,ab,kw

# #3 (major adverse cardiovascular event OR adverse cardiovascular event OR cardiovascular event OR cardiovascular outcome OR MACE):ti,ab,kw

# #4 (prediction model OR risk stratification OR risk score OR risk assessment):ti,ab,kw

# #5 #1 OR #2

# #6 #3 AND #4 AND #5

# Web of Science

# (TS=(Percutaneous Coronary Intervention) OR AB=(Coronary Intervention, Percutaneous OR Coronary Interventions, Percutaneous OR Intervention, Percutaneous Coronary OR Interventions, Percutaneous Coronary OR Percutaneous Coronary Interventions OR Percutaneous Coronary Revascularization OR Coronary Revascularization, Percutaneous OR Coronary Revascularizations, Percutaneous OR Percutaneous Coronary Revascularizations OR Revascularization, Percutaneous Coronary OR Revascularizations, Percutaneous Coronary)) AND (AB=(major adverse cardiovascular event OR adverse cardiovascular event OR cardiovascular event OR cardiovascular outcome OR MACE) )AND (AB= (prediction model OR risk stratification OR risk score OR risk assessment))

# Supplementary Table 2. PROBAST Signaling questions

# 1. Participants

# 1.1. Were appropriate data sources used, e.g., cohort, RCT, or nested case-control study data?

# 1.2. Were all inclusions and exclusions of participants appropriate?

# 2. Predictors

# 2.1. Were predictors defined and assessed in a similar way for all participants?

# 2.2. Were predictor assessments made without knowledge of outcome data?

# 2.3. Are all predictors available at the time the model is intended to be used?

# 3. Outcome

# 3.1. Was the outcome determined appropriately?

# 3.2. Was a prespecified or standard outcome definition used?

# 3.3. Were predictors excluded from the outcome definition?

# 3.4. Was the outcome defined and determined in a similar way for all articipants?

# 3.5. Was the outcome determined without knowledge of predictor information?

# 3.6. Was the time interval between predictor assessment and outcome determination appropriate?

# 4. Analysis

# 4.1. Were there a reasonable number of participants with the outcome?

# 4.2. Were continuous and categorical predictors handled appropriately?

# 4.3. Were all enrolled participants included in the analysis?

# 4.4. Were participants with missing data handled appropriately?

# 4.5. Was selection of predictors based on univariable analysis avoided?

# 4.6. Were complexities in the data (e.g., censoring, competing risks, sampling of control participants) accounted for appropriately?

# 4.7. Were relevant model performance measures evaluated appropriately?

# 4.8. Were model overfitting, underfitting, and optimism in model performance accounted for?

# 4.9. Do predictors and their assigned weights in the final model correspond to the results from the reported multivariable analysis?

# Supplementary Appendix 3. TRIPOD Checklist

| **Section/Topic** | **Item** | **Development or Validation?** | **Checklist Item** |
| --- | --- | --- | --- |
| **Title and abstract** |  |  |  |
| Title | 1 | D;V | Identify the study as developing and/or validating a multivariable prediction model, the target population, and the outcome to be predicted. |
| Abstract | 2 | D;V | Provide a summary of objectives, study design, setting, participants, sample size, predictors, outcome, statistical analysis, results, and conclusions. |
| **Introduction** |  |  |  |
| Background and objectives | 3a | D;V | Explain the medical context (including whether diagnostic or prognostic) and rationale for developing or validating the multivariable prediction model, including references to existing models. |
|  | 3b | D;V | Specify the objectives, including whether the study describes the development or validation of the model, or both. |
| **Methods** |  |  |  |
| Source of data | 4a | D;V | Describe the study design or source of data (e.g., randomized trial, cohort, or registry data), separately for the development and validation datasets, if applicable. |
|  | 4b | D;V | Specify the key study dates, including start of accrual; end of accrual; and, if applicable, end of follow-up |
| Participants | 5a | D;V | Specify key elements of the study setting (e.g., primary care, secondary care, general population) including number and location of centres |
|  | 5b | D;V | Describe eligibility criteria for participants. |
|  | 5c | D;V | Give details of treatments received, if relevant. |
| Outcome | 6a | D;V | Clearly define the outcome that is predicted by the prediction model, including how and when assessed. |
|  | 6b | D;V | Report any actions to blind assessment of the outcome to be predicted. |
| Predictors | 7a | D;V | Clearly define all predictors used in developing the multivariable prediction model, including how and when they were measured |
|  | 7b | D;V | Report any actions to blind assessment of predictors for the outcome and other predictors. |
| Sample size | 8 | D;V | Explain how the study size was arrived at. |
| Missing data | 9 | D;V | Describe how missing data were handled (e.g., complete-case analysis, single imputation, multiple imputation) with details of any imputation method. |
| Statistical analysis methods | 10a | D | Describe how predictors were handled in the analyses. |
|  | 10b | D | Specify type of model, all model-building procedures (including any predictor selection), and method for internal validation. |
|  | 10c | V | For validation, describe how the predictions were calculated. |
|  | 10d | D;V | Specify all measures used to assess model performance and, if relevant, to compare multiple models. |
|  | 10e | V | Describe any model updating (e.g., recalibration) arising from the validation, if done. |
| Risk groups | 11 | D;V | Provide details on how risk groups were created, if done. |
| Development vs. validation | 12 | V | For validation, identify any differences from the development data in setting, eligibility criteria, outcome, and predictors. |
| **Results** |  |  |  |
| Participants | 13a | D;V | Describe the flow of participants through the study, including the number of participants with and without the outcome and, if applicable, a summary of the follow-up time. A diagram may be helpful. |
|  | 13b | D;V | Describe the characteristics of the participants (basic demographics, clinical features, available predictors), including the number of participants with missing data for predictors and outcome. |
|  | 13c | V | For validation, show a comparison with the development data of the distribution of important variables (demographics, predictors, and outcome). |
| Model development | 14a | D | Specify the number of participants and outcome events in each analysis. |
|  | 14b | D | If done, report the unadjusted association between each candidate predictor and outcome. |
| Model specification | 15a | D | Present the full prediction model to allow predictions for individuals (i.e., all regression coefficients, and model intercept or baseline survival at a given time point). |
|  | 15b | D | Explain how to use the prediction model. |
| Model performance | 16 | D;V | Report performance measures (with CIs) for the prediction model. |
| Model updating | 17 | V | If done, report the results from any model updating (i.e., model specification, model performance). |
| **Discussion** |  |  |  |
| Limitations | 18 | D;V | Discuss any limitations of the study (such as nonrepresentative sample, few events per predictor, missing data). |
| Interpretations | 19a | V | For validation, discuss the results with reference to performance in the development data, and any other validation data. |
|  | 19b | D;V | Give an overall interpretation of the results, considering objectives, limitations, results from similar studies, and other relevant evidence. |
| Implications | 20 | D;V | Discuss the potential clinical use of the model and implications for future research. |
| **Other information** |  |  |  |
| Supplementary information | 21 | D;V | Provide information about the availability of supplementary resources, such as study protocol, Web calculator, and datasets. |
| Funding | 22 | D;V | Give the source of funding and the role of the funders for the present study |

# Supplementary Appendix 4. PRISMA2020 Checklist

| **Section and Topic** | **Item #** | **Checklist item** | **Location where item is reported** |
| --- | --- | --- | --- |
| **TITLE** | | |  |
| Title | 1 | Identify the report as a systematic review. |  |
| **ABSTRACT** | | |  |
| Abstract | 2 | See the PRISMA 2020 for Abstracts checklist. |  |
| **INTRODUCTION** | | |  |
| Rationale | 3 | Describe the rationale for the review in the context of existing knowledge. |  |
| Objectives | 4 | Provide an explicit statement of the objective(s) or question(s) the review addresses. |  |
| **METHODS** | | |  |
| Eligibility criteria | 5 | Specify the inclusion and exclusion criteria for the review and how studies were grouped for the syntheses. |  |
| Information sources | 6 | Specify all databases, registers, websites, organisations, reference lists and other sources searched or consulted to identify studies. Specify the date when each source was last searched or consulted. |  |
| Search strategy | 7 | Present the full search strategies for all databases, registers and websites, including any filters and limits used. |  |
| Selection process | 8 | Specify the methods used to decide whether a study met the inclusion criteria of the review, including how many reviewers screened each record and each report retrieved, whether they worked independently, and if applicable, details of automation tools used in the process. |  |
| Data collection process | 9 | Specify the methods used to collect data from reports, including how many reviewers collected data from each report, whether they worked independently, any processes for obtaining or confirming data from study investigators, and if applicable, details of automation tools used in the process. |  |
| Data items | 10a | List and define all outcomes for which data were sought. Specify whether all results that were compatible with each outcome domain in each study were sought (e.g. for all measures, time points, analyses), and if not, the methods used to decide which results to collect. |  |
|  | 10b | List and define all other variables for which data were sought (e.g. participant and intervention characteristics, funding sources). Describe any assumptions made about any missing or unclear information. |  |
| Study risk of bias assessment | 11 | Specify the methods used to assess risk of bias in the included studies, including details of the tool(s) used, how many reviewers assessed each study and whether they worked independently, and if applicable, details of automation tools used in the process. |  |
| Effect measures | 12 | Specify for each outcome the effect measure(s) (e.g. risk ratio, mean difference) used in the synthesis or presentation of results. |  |
| Synthesis methods | 13a | Describe the processes used to decide which studies were eligible for each synthesis (e.g. tabulating the study intervention characteristics and comparing against the planned groups for each synthesis (item #5)). |  |
|  | 13b | Describe any methods required to prepare the data for presentation or synthesis, such as handling of missing summary statistics, or data conversions. |  |
|  | 13c | Describe any methods used to tabulate or visually display results of individual studies and syntheses. |  |
|  | 13d | Describe any methods used to synthesize results and provide a rationale for the choice(s). If meta-analysis was performed, describe the model(s), method(s) to identify the presence and extent of statistical heterogeneity, and software package(s) used. |  |
|  | 13e | Describe any methods used to explore possible causes of heterogeneity among study results (e.g. subgroup analysis, meta-regression). |  |
|  | 13f | Describe any sensitivity analyses conducted to assess robustness of the synthesized results. |  |
| Reporting bias assessment | 14 | Describe any methods used to assess risk of bias due to missing results in a synthesis (arising from reporting biases). |  |
| Certainty assessment | 15 | Describe any methods used to assess certainty (or confidence) in the body of evidence for an outcome. |  |
| **RESULTS** | | |  |
| Study selection | 16a | Describe the results of the search and selection process, from the number of records identified in the search to the number of studies included in the review, ideally using a flow diagram. |  |
|  | 16b | Cite studies that might appear to meet the inclusion criteria, but which were excluded, and explain why they were excluded. |  |
| Study characteristics | 17 | Cite each included study and present its characteristics. |  |
| Risk of bias in studies | 18 | Present assessments of risk of bias for each included study. |  |
| Results of individual studies | 19 | For all outcomes, present, for each study: (a) summary statistics for each group (where appropriate) and (b) an effect estimate and its precision (e.g. confidence/credible interval), ideally using structured tables or plots. |  |
| Results of syntheses | 20a | For each synthesis, briefly summarise the characteristics and risk of bias among contributing studies. |  |
|  | 20b | Present results of all statistical syntheses conducted. If meta-analysis was done, present for each the summary estimate and its precision (e.g. confidence/credible interval) and measures of statistical heterogeneity. If comparing groups, describe the direction of the effect. |  |
|  | 20c | Present results of all investigations of possible causes of heterogeneity among study results. |  |
|  | 20d | Present results of all sensitivity analyses conducted to assess the robustness of the synthesized results. |  |
| Reporting biases | 21 | Present assessments of risk of bias due to missing results (arising from reporting biases) for each synthesis assessed. |  |
| Certainty of evidence | 22 | Present assessments of certainty (or confidence) in the body of evidence for each outcome assessed. |  |
| **DISCUSSION** | | |  |
| Discussion | 23a | Provide a general interpretation of the results in the context of other evidence. |  |
|  | 23b | Discuss any limitations of the evidence included in the review. |  |
|  | 23c | Discuss any limitations of the review processes used. |  |
|  | 23d | Discuss implications of the results for practice, policy, and future research. |  |
| **OTHER INFORMATION** | | |  |
| Registration and protocol | 24a | Provide registration information for the review, including register name and registration number, or state that the review was not registered. |  |
|  | 24b | Indicate where the review protocol can be accessed, or state that a protocol was not prepared. |  |
|  | 24c | Describe and explain any amendments to information provided at registration or in the protocol. |  |
| Support | 25 | Describe sources of financial or non-financial support for the review, and the role of the funders or sponsors in the review. |  |
| Competing interests | 26 | Declare any competing interests of review authors. |  |
| Availability of data, code and other materials | 27 | Report which of the following are publicly available and where they can be found: template data collection forms; data extracted from included studies; data used for all analyses; analytic code; any other materials used in the review. |  |

# Supplementary Table 1. Predictors and MACE definitions

| **STUDY** | **Predictors** | **MACE definition** |
| --- | --- | --- |
| **For STEMI patients** | | |
| Ma J-2022 | age, male, Killip classification, plasma DPP4 activity, diabetes-mellitus, smoking history | cardiac death, non-fatal MI, heart failure, or stroke |
|  | age, male, Killip classification, diabetes-mellitus, smoking history |  |
| Cui L-2022 | female, age, anterior wall myocardial infarction, Killip classification, preoperative malignant arrhythmia, systolic blood pressure, LVEF, ischemia time | death, recurrent MI, malignant arrhythmia, severe heart failure, cardiogenic shock |
| Shi S-2022 | age, diabetes mellitus, Scr, CK-MB, hs-CRP, NLR, PLR, PCT, IL-6, LVEF | all-cause mortality, severe arrhythmias, cardiac arrest, angina attacks, and heart failure. |
| Wang Y-2022 | smoking, hypertension, Killip grade≥3, LVESd>40 mm, hs-CRP>10 mg/L | all-cause mortality, non-lethal myocardial infarction, ischemic stroke, severe heart failure (heart failure) receiving heart failure treatment, and late revascularization (>90 days). |
| Zhang X-2022 | NGAL, creatinine, time from AMI to the opening of infarct-related artery, LVEF, infarct-related artery | fatal cardiovascular disease, non-fatal myocardial infarction, non-fatal stroke, and recurrent angina |
| Fang C-2022 | Killip grade II-IV, urea nitrogen, LVEF, and NT-proBNP | death from any cause, recurrent AMI, or ischemic stroke. |
| Marcos-Garcés-2022 | time to reperfusion >4.15 h, GRACE risk score > 155, CMR-LVEF <40%, and MVO >1.5 segments | cardiovascular (CV) death, nonfatal myocardial infarction (NF-MI), or re-admission for acute decompensated heart failure (HF). |
| Yao W -2022 | Female gender, hypertension, in-hospital VA, 3-vessel disease, baseline NT-proBNP, and peak cTnI | cardiac death, myocardial infarction, hospitalization for chest pain or congestive heart failure, late revascularization, or arrhythmia |
| Yu J-2022 | age, history of DM, CRP, and multivessel disease | all-cause death, any myocardial infarction, and any repeat revascularization. |
| Ma Q-2021 | S（5; 5） Contrast, Vertl LngREmph, WavEnLH s | death, occurrence of new congestive heart failure, and myocardial reinfarction |
|  | Contrast, Vertl LngREmph, WavEnLH s, cTnI |  |
| Zhao E-2020 | Apelin-12 change rate, apelin-12 level, age, pathological Q wave, myocardial infarction history, anterior wall myocardial infarction, Killip’s classification > I, uric acid, total cholesterol, cTnI, the left atrial diameter | cardiac death, clinically driven target lesion revascularization, recurrent target vessel myocardial infarction, cardiogenic shock, or demonstrated congestive heart failure |
|  | age, pathological Q wave, myocardial infarction history, anterior wall myocardial infarction, Killip’s classification > I, uric acid, total cholesterol, cTnI, ,the left atrial diameter |  |
| Zhao X-2020 | age, history of hypertension, history of chronic kidney disease, EF, TBil, stent implantation, Killip classification, use of tirofiban during PCI, coronary revascularization at admission, use of ACEIs, use of beta-blockers | all-cause mortality, cardiac cause death, recurrent myocardial infarction(MI), and ischemic stroke |
| **For AMI/MI patients** | | |
| Li Q-2022 | ST-segment deviation, diabetes, LVEF, eGFR, Hb | all-cause death, non-fatal MI, non-fatal stroke, malignant arrhythmia, new heart failure or admission for heart failure, unplanned revascularization |
|  | ST-segment deviation, diabetes, eGFR, Hb |  |
|  | ST-segment deviation, diabetes, Hb |  |
| Zeng W-2022 | diabetes, TnI, LDL, LP(a), eGFR, 3-vessel disease | new or recurrent AMI, unstable angina, new-onset stroke, cardiac death, unplanned repeat PCI, heart failure, or any hospitalization due to cardiac causes. |
| Cao J-2021 | residual inflammatory risk, Hb1Ac, leukocyte count, LVEF, NT-proBNP | pump failure, cardiogenic shock, malignant arrhythmia, and death |
| Pan D -2021 | age, diabetes mellitus, LDL-C level, LP(a) level, LVEF, Syntax score, hypersensitive troponin T | cardiac death, recurrent AMI, unplanned revascularization, or rehospitalization for any cardiovascular disease, including heart failure, nonfatal ischemic stroke, or unstable angina |
| Wu C-2021 | age, sex, left ventricular ejection fraction, Killip class, systolic blood pressure, creatinine, white blood cell count, heart rate and blood glucose | all-cause death, recurrent AMI, or non-fatal ischemic stroke |
| Zhao X -2020 | age, a history of diabetes mellitus, AF, CKD,CABG, the Killip classification, ejection fraction at admission, hs-CRP level, eGFR, d-dimer level, multivessel lesions, the culprit vessel | all-cause mortality, cardiac mortality, MI recurrence, and stroke (ischemic stroke). |
| **For ACS patients** | | |
| Huang G-2022 | Gensini score, platelet count, PLR, cTnI, BNP | acute heart failure, severe arrhythmia, non-fatal myocardial infarction, stroke, angina recurrence, and death |
| Li Y-2022 | age, smoking, WBC, NT proBNP, Scr, LVEF | recurrent angina, congestive heart failure, repeat revascularization, non-fatal myocardial infarction, severe arrhythmias, and cardiac death. |
| Kong S -2021 | lactate level, age, left anterior descending branch stenosis, right coronary artery stenosis, BNP level, LVEF | all-cause mortality, clinically driven re-vascularization of target lesions, new or recurrent myocardial infarction, and stroke. |
| **For other patients** | | |
| Grayson AD-2006 | advanced age, female sex, cerebrovascular disease, cardiogenic shock, priority, treatment of the left main stem, graft lesions during PCI | in-hospital mortality, Q-wave myocardial infarction, emergency coronary artery bypass graft surgery, and cerebrovascular accidents |
| He H-2023 | smoking, history of hypertension, 3-vessel disease, FBG, high CK-MB level, low LVEF | malignant arrhythmias, non-fatal reinfarction, new-onset acute heart failure, and all-cause or cardiac death. |
| Abbreviations: NGAL, neutrophil gelatinase-associated lipocalin. hs-CRP, the high-sensitivity C-reactive protein. LVEF, left ventricular ejection fraction. eGFR, the estimated glomerular filtration rate. AIC, Akaike information criterion. BNP, brain natriuretic peptide. ACEIs, angiotensin-converting enzyme inhibitors. AF, atrial fibrillation. CKD, chronic kidney disease. CABG, coronary artery bypass grafting. | | |

# Supplementary Table 2. PROBAST results of each study

|  | **participants** | | **predictors** | | | **results** | | | | | | **analysis** | | | | | | | | |
| --- | --- | --- | --- | --- | --- | --- | --- | --- | --- | --- | --- | --- | --- | --- | --- | --- | --- | --- | --- | --- |
| Studies | 1 | 2 | 3 | 4 | 5 | 6 | 7 | 8 | 9 | 10 | 11 | 12 | 13 | 14 | 15 | 16 | 17 | 18 | 19 | 20 |
| Ma J-2022 | N | N | Y | NI | Y | N | N | Y | Y | NI | Y | N | Y | Y | N | N | NI | Y | N | NI |
| Li Q-2022 | N | N | Y | NI | Y | N | N | N | Y | NI | Y | Y | Y | Y | N | Y | NI | N | N | Y |
| Huang G-2022 | N | N | Y | NI | Y | N | N | Y | Y | NI | N | N | Y | Y | N | N | NI | N | N | Y |
| Cui L-2022 | N | N | Y | NI | Y | N | N | N | Y | NI | PN | N | N | Y | N | Y | NI | Y | N | PY |
| Cao J-2021 | N | N | Y | NI | Y | N | N | N | Y | NI | N | N | PY | Y | N | Y | NI | N | N | PY |
| Zeng W-2022 | N | N | Y | NI | Y | N | N | Y | Y | NI | Y | N | N | Y | N | N | NI | N | N | N |
| Li Y-2022 | N | N | Y | NI | Y | N | N | N | Y | NI | N | N | N | Y | N | Y | NI | Y | N | NI |
| Shi S-2022 | N | N | Y | NI | Y | N | N | N | Y | NI | N | N | N | Y | N | N | NI | Y | N | PY |
| Wang Y-2022 | N | N | Y | NI | N | N | N | Y | Y | NI | PN | Y | Y | Y | N | N | NI | Y | N | PY |
| Zhang X-2022 | N | N | Y | NI | Y | N | N | Y | Y | NI | Y | N | N | Y | N | Y | NI | N | Y | PY |
| He H-2023 | N | N | Y | NI | Y | N | N | N | Y | NI | Y | N | NI | Y | N | N | NI | N | N | Y |
| Grayson AD-2006 | N | NI | Y | NI | Y | N | N | Y | Y | NI | Y | NI | N | Y | Y | Y | NI | Y | N | Y |
| Zhao E-2020 | N | NI | Y | NI | Y | N | N | Y | Y | NI | Y | Y | N | Y | N | Y | NI | Y | Y | NI |
| Zhao X-2020 | N | NI | Y | NI | N | N | N | Y | Y | NI | Y | Y | PN | Y | N | Y | NI | Y | N | NI |
| Zhao X-2020 | N | NI | Y | NI | Y | N | N | Y | Y | NI | Y | Y | N | Y | N | Y | NI | N | N | NI |
| Kong S-2021 | Y | N | Y | Y | Y | N | N | Y | Y | NI | Y | Y | N | Y | N | Y | NI | Y | N | Y |
| Ma Q-2021 | N | N | Y | NI | Y | N | N | Y | Y | NI | Y | N | Y | Y | N | Y | NI | Y | N | NI |
| Pan D-.2021 | Y | N | Y | NI | Y | N | N | Y | Y | NI | Y | Y | Y | Y | N | N | NI | Y | N | NI |
| Wu C-2021 | N | Y | Y | NI | Y | N | N | Y | Y | NI | Y | Y | N | Y | Y | Y | Y | Y | Y | Y |
| Fang C-2022 | N | N | Y | NI | Y | N | N | Y | Y | NI | Y | Y | Y | Y | N | Y | NI | Y | N | PY |
| Marcos-Garcés-2022 | Y | NI | Y | Y | Y | N | N | Y | Y | NI | Y | Y | N | Y | NI | N | N | N | N | Y |
| Yao W-2022 | N | N | Y | NI | Y | N | N | Y | Y | NI | Y | N | Y | Y | NI | Y | Y | Y | N | PY |
| Yu J-2022 | N | Y | Y | NI | Y | N | N | Y | Y | NI | Y | NI | N | Y | NI | Y | NI | N | N | NI |
| Abbreviations: N, no. Y, yes. NI, no information. | | | | | | | | | | | | | | | | | | | | |

# Supplementary Figure 1. TRIPOD results of each study

##
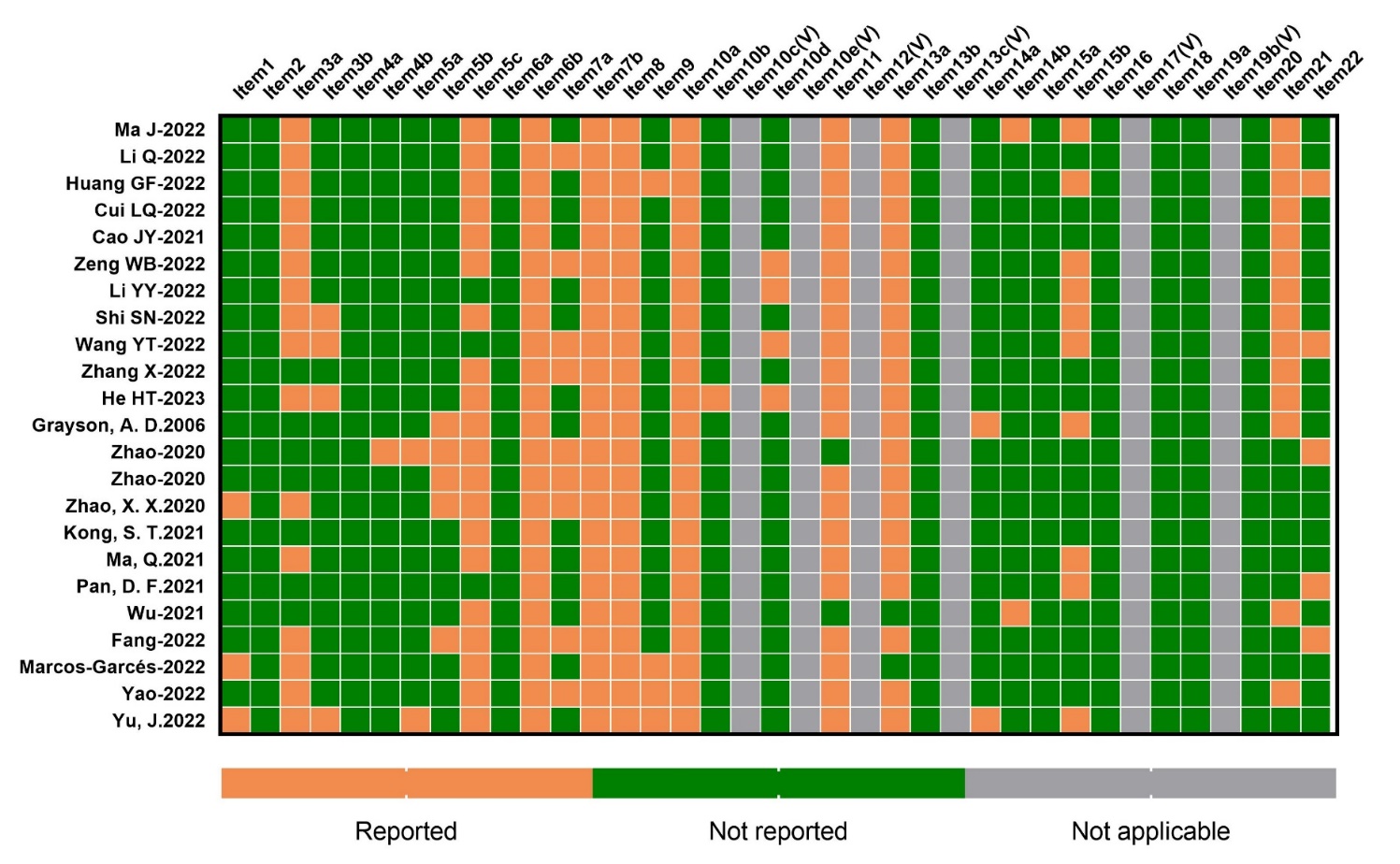

Supplement: Supplementary file 1 [file Datasheet1.docx]
